# SkQ1 treatment and food restriction — two ways to retard an aging program of organisms

**DOI:** 10.18632/aging.100410

**Published:** 2011-12-12

**Authors:** Vladimir P. Skulachev

**Affiliations:** Belozersky Institute of Physico-Chemical Biology, Lomonosov Moscow State University, Moscow 119991, Russia

**Keywords:** Aging, mitochondria, food (caloric) restriction, SkQ1, mitochondria-targeted antioxidant

## Abstract

Effects of the mitochondria-targeted antioxidant SkQ1 and food restriction are compared. In both cases there is a remarkable increase in the median lifespan of organisms belonging to many different taxonomic ranks. Essentially, both SkQ1 treatment and restriction in food intake retard development of numerous adverse traits of senescence. This relationship could be predicted assuming that SkQ1 and food restriction inhibit the execution of an aging program. It is hypothesized that food restriction is perceived by organisms as a signal of starvation, which can be catastrophic for the population. Under these conditions, the organism switches off an aging program that is favorable for evolvability of the species but counterproductive for the individual. Unfortunately, food restriction is accompanied by some other effects, e.g., constant anxiety and attempts to scan as large a space as possible looking for food. Such side effects seem to be absent in the case of inhibition of the aging program by SkQ1.

As geroprotectors, SkQ1 and food restriction are similar in two very important aspects. Both prolong lifespan in many species and retard development of numerous traits of aging [cf. 1-5 and 6, 7]. Furthermore, there is an indication that the food restriction effect includes at least one component characteristic of the mechanism of action of SkQ1, i.e. antioxidant activity at the mitochondrial level. Fernandes and coauthors [8] found that 40% caloric restriction resulted in a 10-fold increase in the lifespan of C57Bl/6 mice poisoned by the prooxidant paraquat, a cation with delocalized charge, which is targeted to mitochondria in its reduced form (I. I Severina et al., in preparation) 1.

The effect of food restriction on longevity was described in 1934, when McCay and coworkers [10] found that it extended the lifespan of rats (see also [11, 12]). The restriction was imposed at early stages of life and initially resulted in retardation of growth. When the food restriction was terminated, the animals rapidly increased in size to reach the average but lived longer by 70% (males) and 48% (females) than rats that were fed *ad libitum* over their whole life (cf. our data on a larger effect of SkQ1 on longevity in the mouse and mole-vole males than females [1]). A large decrease in death rates from pulmonary diseases and certain tumors was observed (again similar to SkQ1 [2, 3, 13]). It was also noted that food-restricted animals appeared active and young irrespective of their actual age (another similarity with SkQ1 [2, 3]).

Later, the positive effect of a certain food restriction on lifespan was demonstrated on a great variety of organisms — from yeast to rhesus monkeys and humans [6, 7, 14, 15]. With the appearance of Harman's hypothesis on the role of ROS in aging [16], this effect came to be explained in terms of a decreased volume of food being oxidized by oxygen and, as a consequence, a decreased production of ROS [17]. That this assumption was invalid was evident already in early works on dietary restriction, when Will and McCay [18] reported that the heat production per kg body weight in the food-restricted rat cohort was *higher* rather than lower than in the control. Further research gave direct evidence against this commonly accepted viewpoint. First, it was found that for drosophila it is enough to starve for only the two first days of life to become long-lived to the same extent as if the flies were subjected to dietary restriction over their entire life [19] (similar relationships were revealed with SkQ1 [2]). Second, not only an excess of food, but also its smell attenuated the geroprotecting effect provided by food restriction on drosophila and *C. elegans* [20, 21]. These observations are unlikely to be specific for invertebrates. As far back as 1934, Robertson et al. [22] discovered in their experiments on mice that a two-day “fasting” every week is sufficient for their lifespan to be extended. Carr et al. [23] observed, again with mice, that reproductive ability lost by the end of the first year in the case of unrestricted feeding was preserved until at least the 21st month of life when the mice were limited in food ration over the first 11-15 months and then received food *ad libitum* (cf. SkQ1-induced fecundity increase of mice [2, 3] and mole-vole [1]). According to data of Stuchlikova et al. [24], rats, mice, and golden hamsters restricted in food ration by 50% over two years lived 20% longer than the controls. Dietary restriction during the first year of life prolonged the lifespan by 40-60%, and during the second year by only 30-40%.

Further research showed that the effect of dietary restriction involves both the carbohydrate and protein components of food. The effect of proteins restriction is associated with only one amino acid, viz. methionine [25-28]. Methionine is an essential amino acid that is not synthesized in mammals, so food is the only source of this compound. It was found that a diet in which proteins are replaced by a mixture of amino acids containing no methionine not only favors a longer life but decreases mitochondrial ROS generation and oxidative damage to *mitochondrial* DNA [27, 28]. Interestingly, dietary restriction has no effect on the oxidation of *nuclear* DNA [29].

In our opinion [5], food restriction is perceived by an organism as a worrying signal of food shortage. As known, starvation entails decline of many physiological functions, and in particular decreased fecundity [6]. And this, in turn, jeopardizes the very existence of the population. To prevent, at least in part, such turn of events, it might be sufficient to cancel the aging program, thereby prolonging the reproductive period of the individual and thus increasing the total number of progeny. If this is the case, the impact of dietary restriction on lifespan is only indirectly related to ROS, rather being a regulatory effect. That is why such evidently signaling effects as a short-term fasting (or, vice versa, smell of food), rather than food shortage (or excess) over the entire life, exert a powerful effect on the life cycle parameters. It is remarkable that a *temporary* dietary restriction (fasting) is better than a constant restriction. The most probably, the signal of food shortage can be given for a fairly short time, whereas starving for a long time is harmful for an organism.

The signaling nature of the effect of dietary restriction provides a good rationale for the results of experiments with methionine. Apparently, the organism determines the amount of available food (and, first of all, essential amino acids required for protein synthesis) by monitoring the level of only one of the amino acids, specifically methionine.

Significantly, dietary restriction not only extends lifespan, but also prolongs youth, as already mentioned by McCay, the discoverer of this phenomenon [10]. Quite illustrative in this respect is the recently reported results of studies by Weindruch and his group on primates [14]. Twenty-year-long experiments on 76 macaques (started when the animals were from 7 to 14 years old) showed that a long-term 30% dietary restriction has the following effects: (1) a sharp decrease in age-related death rate (over 30 years, 20% against 50% in the control group fed *ad libitum*), (2) exclusion of diabetes from the causes of death, (3) halving the death rate from cancer (in macaques this is primarily intestine adenocarcinoma), (4) decrease in the death rate induced by cardiovascular diseases, (5) decrease in osteoporosis, (6) arrest of the development of such age-related traits as sarcopenia, decline in brain gray matter, alopecia, canities, etc. It is noteworthy that the majority of these effects are also characteristic of the action of SkQ1 [1-4]. By the age of 30 years, 80% of the surviving control macaques showed some traits of aging, whereas in the experimental group such traits were observed in only about 20% of the animals.

This experiment on monkeys is still far from completion, and, therefore, we can say nothing about the effect of dietary restriction on the maximal lifespan of primates. However, some data of this kind are available for rodents [12, 24]. Here it was found that the median lifespan in mice and hamsters increases much more markedly than the maximal lifespan (another similarity with the effect of SkQ1 [1-3]). The simplest mechanistic explanation of this phenomenon is arrest (or at least retardation) of an aging program. Therewith, other ontogenetic programs, first of all body growth, can also be retarded. These phenomena are observed with a rather severe and long-term fasting [10]. However, a more moderate dietary restriction can prolong life without inhibiting growth [30].

This situation illustrates the very fact that moderate and severe starvations have opposite effects on physiological functions. Perhaps this explains contradictory data concerning the effect of food restriction on skeletal muscles and the immune system. On one hand, some authors state that it adversely affects both the muscle system and immunity^2^. On the other hand, Weindruch and coworkers [14] reported the lack of sarcopenia in food-restricted monkeys, and McCay and coworkers [10] observed resistance to pulmonary diseases in rats subjected to dietary restriction (cf. deceleration of age-dependent involution of the thymus and follicular spleen compartments in rats administered SkQ1 [3, 35])^3^.

The fact that dietary restriction adversely affects some vitally important parameters is not surprising. Animals usually do not tend to overeat even if they are not restricted in access to food. They normally eat as much as needed for healthy functioning. Therefore, long-term dietary restriction entails some disorders in vital functions. It is also clear that such disorders are more probable the longer is starvation. We already mentioned that long-term and continuous dietary restriction is not at all necessary for the geroprotecting effect. This explains the controversy in data on the effect of dietary restriction on lifespan as well as on state of the organism. In cases when dietary restriction was not too severe and not too long, positive effects were observed, whereas when gerontologists employed too severe restriction, unfavorable side effects occurred. Thus, it is commonly accepted that long-term dietary restriction decreases the frequency of estrous cycles (sometimes until they cease completely) [31], but in 1949 Carr and coworkers showed that *temporary* dietary restriction prolonged estrous cycles and favored their preservation until extreme old age [23]. (The same effect was observed with SkQ1 [1, 3]). In principle, there is no need to starve over the whole life if starvation is a signal to switch off an aging program. However, there is a probability that too weak or delayed dietary restriction will only partially retard the program, and the geroprotecting effect will be weak.

Another circumstance should be taken into account when considering under-eating as a geroprotector for humans. Actually, if dietary restriction is a signal to warn about starvation, then the organism should respond not only by prolonging life to compensate for the decay of fecundity in lean years. Other responses are also quite possible, and some of them may prove not as attractive as extension of healthy life. For example, it was noted that a hungry mouse, once in a squirrel wheel, does not want to leave it and travels from 6 to 8 km overnight (with normal feeding this distance is always shorter than 1 km) [31]. Obviously, this effect is not consistent with starvation-induced exhaustion and muscle weakness. More likely, we deal here with another response to the starvation signal: extreme anxiety and the attempt to scan as large a space as possible.

It should be mentioned in this context that SkQ1 does not influence the food intake by animals receiving it [1]. Measurement of motivity of 15-20-month-old outbred mice and 129/sv mice showed that 5-250 nmol SkQ1/kg per day decreased rather than increased this parameter. In fact, the old control animals looked more agitated than the SkQ1-treated mice of the same age [36]. An impression arises that SkQ is a “purer” way, when compared with starvation, to retard an aging program, not being accompanied by undesirable side effects.

Various strains of inbred mice may differ greatly in lifespan, which sometimes varies threefold [37, 38]. It seems possible that such a difference is sometimes due to mutations affecting execution of an aging program. In turn, food restriction, if it really inhibits an aging program, should be inefficient in strains already lacking such a program or mechanisms of food intake sensing. Recently, Sohal and coworkers compared C57Bl/6 mice (that respond to starvation with an increase in lifespan) and DBA/2 mice that do not show such an increase [39]. Metabolic characteristics of DBA/2 mice were found to point to partial uncoupling of mitochondrial respiration and ADP phosphorylation, an effect strongly lowering mitochondrial ROS production in the resting state [39, 40]. Perhaps long-lived 129/sv mice, which are insensitive to SkQ1 with respect to lifespan and show regular estrous cycles for as long as 20 months even without SkQ1 [1], are similar to DBA/2 mice. Remarkably, 129/sv and DBA/2 mice show similar patterns of age-dependent changes of some gene transcripts, differing in this respect from BALB/c. However, C57Bl/6 mice, a line responding to food restriction, resemble DBA/2 if this criterion was applied [40]. Quite recently, Hempenstall et al. [41] reported that food restriction-sensitive C57Bl/6 mice and insensitive DBA/2 mice oppositely responded to fasting when the blood glucose level was measured. In the first case, glucose level *decreased*, whereas in the latter case it *increased*. Blood glucose looks as a good candidate for a diet component to monitor food level of carbohydrates, just as blood methionine is such a component to monitor the level of proteins. If this is the case, it is hardly surprising that DBA/2 mice fail to recognize food restriction as a signal for a starvation period. It seems quite possible that mice of various lines differ in the threshold level of food restriction when diet limitation turns from a starvation signal to a harmful deficit of dietary components. This is never taken into account in caloric restriction studies. Apparently, commonly accepted 40% food restriction is close to such a threshold, thus resulting in great variation of responses of different strains of animals. The present situation in this field is exemplified by a recent publication of Liao at al. [38] who analyzed responses of 41 recombinant inbred strains of mice to 40% food restriction. For females, 8 lines responded to such restriction by an increase in lifespan, 10 by its decrease, and 21 showed no statistically reliable response. For males, these values were 2, 28, and 11. Unfortunately, each group was composed of only five animals living in one cage. For sure, food restriction worsened social climate in such small communities, resulting in increase in deviation of the lifespan data. In fact, in *ad libitum* groups this parameter varied from 407 to 1208 days for females and from 504 to 1152 days for males, whereas in food restricted groups it was 113 – 1225 days for females and 217-1215 days for males.

Another disadvantage of the abovementioned approach is that the most marked effect of food restriction is an increase in healthspan rather than lifespan. Large lifespan increase by switching off the aging program can be observed provided that ambient conditions are not artificially improved by, say, abolishing infection (a non-SPF vivarium or outdoor cages are better than an SPF vivarium). For further increase in lifespan, cancer must be somehow excluded. Both SkQ1 and food restriction are effective in retarding development of only a limited number of cancers, whereas others are resistant to these factors. Quite recently an example of a rodent cancelling its aging program and, moreover, solving the problem of cancer was described. We mean the naked mole-rat (*Heterocephalus glaber*). This animal of mouse size lives ten times longer than mice. The probability of its death does not increase with age [42], indicating that an aging program is not operating. The program seems to be switched off somewhere downstream of mitochondrial ROS since both the rate of ROS generation by resting mitochondria [43] and level of peroxidation of cellular biopolymers [44, 45] in the naked mole rats are *higher* than in mice. The latter finding is consistent with the observation that one of the major antioxidant enzymes, glutathione peroxidase, is 70-fold *less* active in naked mole-rats than in mice [46]. The key for understanding this situation seems to be the observation that H_2_O_2_ fails to induce apoptosis in cultured arteria of naked mole rats [47]. Age-related diseases are not known for naked mole-rats. And cancer has never been observed in naked mole-rats, necropsies failing to reveal tumors [42]. This is apparently due to at least two additional lines of anticancer defense lacking in other rodents. Seluanov et al. reported [48] that naked mole-rat cells in culture express unusually high levels of the tumor suppressor p16^INKYA^, a protein responsible for contact inhibition of growth of cultured cells. Later Liang et al. showed [49] that oncogene expression in the naked mole-rat cells failed to produce cells competent in tumor expression when transferred to immunodeficient mice. The reason for such a failure is that these cells rapidly entered crisis, as evidenced by appearance of anaphase bridges, giant cells with enlarged nuclei, multinuclear cells, and cells with large number of chromosomes or abnormal chromatin. Crisis was also observed after >40 cell doublings of the naked mole-rat cells expressing an oncogene. Crisis in culture was prevented by additional infection of the cells with a retrovirus encoding telomere reverse transcriptase (hTERT). The authors suggested that such activity of hTERT is due to one of its extra-telomeric effects requiring an intracellular hTERT concentration higher than is normally present in naked mole-rat cells.

Apparently, the specific features of naked mole-rats preventing cancer are absent in other studied animals. Therefore, lifespan increase due to interruption of the aging program in these animals is not so strong as in naked mole-rats, since they die as a result of development of those types of cancer which are resistant to food restriction, SkQ1, or other geroprotectors that have been tested. The mole-vole (*Ellobius talpinus*) studied in our group [1] might represent an exception to this rule. As *post mortem* analysis showed, these rodents die almost exclusively due to infections or other non-cancer pathologies and live very much longer when treated with SkQ1. The effect of caloric restriction on mole voles is now being investigated.

## Footnotes

1 Concerning decrease in mitochondrial ROS level by food restriction, see review by Gredilla and Barja [9]

2 Fernandes and coworkers [21] noted that dietary restriction, especially at a young age, inhibits interleukin secretion by macrophages as a response to bacterial polysaccharides. In turn, this adversely affects resistance to sepsis and peritonitis [21, 31]. According to Gardner [32, 33], partial starvation abolishes resistance to influenza virus. For reviews, see Refs. [31, 34].

3 As mentioned above, the effect of SkQ1 on longevity was especially strong in non-SPF vivaria. Apparently, a similar situation is inherent in the food restriction effect. Remarkably, the magnitude of the lifespan increase by food restriction was usually much stronger in experiments published in 1934-1975 (i.e. when SPF practice was unknown or not so widespread) than after 1975.

## Abbreviations

ROS: reactive oxygen species
SkQ1: plastoquinonyl-10(6′-decyltriphenyl)phosphonium
